# P-REX1-Independent, Calcium-Dependent RAC1 Hyperactivation in Prostate Cancer

**DOI:** 10.3390/cancers12020480

**Published:** 2020-02-19

**Authors:** Martin J. Baker, Martín C. Abba, Rafael Garcia-Mata, Marcelo G. Kazanietz

**Affiliations:** 1 Department of Systems Pharmacology and Translational Therapeutics, Perelman School of Medicine, University of Pennsylvania, 1256 Biomedical Research Building II/III, 421 Curie Blvd., Philadelphia, PA 19104-6160, USA; Martin.Baker@pennmedicine.upenn.edu; 2 Centro de Investigaciones Inmunológicas Básicas y Aplicadas, Universidad Nacional de La Plata, CP1900 La Plata, Argentina; mcabba@gmail.com; 3 Department of Biological Sciences, The University of Toledo, Toledo, OH 43606, USA; rafael.garciamata@utoledo.edu

**Keywords:** Rac1, P-Rex1, Rac-GEF, calcium, prostate cancer cells

## Abstract

The GTPase Rac1 is a well-established master regulator of cell motility and invasiveness contributing to cancer metastasis. Dysregulation of the Rac1 signaling pathway, resulting in elevated motile and invasive potential, has been reported in multiple cancers. However, there are limited studies on the regulation of Rac1 in prostate cancer. Here, we demonstrate that aggressive androgen-independent prostate cancer cells display marked hyperactivation of Rac1. This hyperactivation is independent of P-Rex1 activity or its direct activators, the PI3K product PIP_3_ and Gβγ subunits. Furthermore, we demonstrate that the motility and invasiveness of PC3 prostate cancer cells is independent of P-Rex1, supporting the analysis of publicly available datasets indicating no correlation between high P-Rex1 expression and cancer progression in patients. Rac1 hyperactivation was not related to the presence of activating Rac1 mutations and was insensitive to overexpression of a Rac-GAP or the silencing of specific Rac-GEFs expressed in prostate cancer cells. Interestingly, active Rac1 levels in these cells were markedly reduced by elevations in intracellular calcium or by serum stimulation, suggesting the presence of an alternative means of Rac1 regulation in prostate cancer that does not involve previously established paradigms.

## 1. Introduction

Prostate cancer is the most frequently diagnosed malignancy in men in the United States aside from skin cancer, with an estimated 175,000 new cases in 2019, and the second leading cause of cancer-related deaths in men [1]. The 5-year survival rate for patients with localized prostate cancer is >99%. However, this is dramatically reduced to 30% in patients with metastatic prostate cancer [1]. Aggressive metastatic phenotypes are associated with androgen independence, resulting in resistance to androgen ablation or castration therapies (“Castration-Resistant Prostate Cancer” or CRPC). Indeed, prostate cancer cells that have acquired androgen independence have a significant propensity to metastasize. There is currently no treatment available to cure such androgen-independent prostate cancer, and a greater understanding of the mechanisms that drive CRPC is therefore essential [2].

The small GTPases Rac1 and Cdc42 have been recognized as major players in cancer progression and perform essential roles in metastasis [3,4,5]. These G-proteins are members of the Rho-GTPase family, and as such they are binary switches that cycle between an active GTP-bound state and an inactive GDP-bound state. The activation of these GTPases occurs through the action of Guanine nucleotide Exchange Factors (GEFs), which stimulate the release of GDP and therefore allow the subsequent binding of GTP. Conversely, GTPases are deactivated by the hydrolysis of GTP to GDP, which is accelerated by GTPase Activating Proteins (GAPs) [6]. A third class of regulatory proteins, called RhoGDIs, also exert influence on the Rho-GTPase activation cycle, in this case by binding the inactive form and sequestering it in the cytosol [7]. When active, these GTPases and their effectors regulate the generation of actin-rich membrane protrusions that play important roles in cell migration and invasion, with Rac1 driving lamellipodia formation and Cdc42 driving filopodia formation [5,8]. Furthermore, Rac1 has been shown to have additional functions that contribute to cancer progression, including ROS formation [9,10], proliferation [4], gene expression [11,12] and ribosomal biogenesis [13,14].

The dysregulation of Rac1 activation has been reported in multiple diseases, including cancer [3,4,5]. Oncogenic signaling by Rac1 can occur from a mutation, as seen in melanoma with the Rac1^P29S^ mutant [15,16], or alternatively from dysregulation of the activation cycle. This includes enhanced activation of membrane receptors or their effectors (e.g., PI3K), up-regulation/mutations of Rac-GEFs, or down regulation of Rac-GAPs [3,6,17,18]. Elevated Rac1 activation has been largely associated with the progression of various cancers and metastatic dissemination of cancer cells. Specifically, for prostate cancer, Rac1 signaling has been implicated in androgen receptor signaling leading to proliferation [19,20] and epithelial-to mesenchymal transition (EMT) [21,22]. Recent work has also demonstrated that the spatial regulation of Rac1 activation in prostate cancer cells, an essential component of directed cell migration, is dependent on RhoH signaling [23].

The potential involvement of Rac-GEFs in prostate cancer has received little attention. There are two families of Rac-GEFs, the dbl-like GEFs and the DOCK GEFs. One of the few Rac-GEFs characterized in prostate cancer is P-Rex1, a dbl-like exchange factor originally identified in neutrophils and later found to be involved in the progression of specific cancer types such as breast cancer and melanoma [24,25,26,27,28]. P-Rex1 nucleotide exchange activity is dependent on the synergistic stimulation by the PI3K product PIP_3_ and Gβγ subunits released from activated heterotrimeric G-proteins [24]. Consistent with this regulation, P-Rex1 is an effector of tyrosine-kinase receptors (e.g., EGFR, ErbB3 and IGF-IR) and GPCRs (e.g., CXCR4 and FPR1), mediating Rac1-dependent migratory responses in response to their stimulation [26,29,30]. Importantly, P-Rex1 overexpression has been shown in various cancer types, particularly luminal breast cancer and prostate cancer [26,28]. It has been reported that P-Rex1 is highly expressed in PC3 prostate cancer cells relative to androgen-dependent cell lines, and that silencing P-Rex1 in PC3 prostate cancer cells leads to a reduction in activated Rac1, as well as in the migratory and invasive capacity of these cells [28].

Towards the goal of better understanding the relevance of Rac1 signaling in prostate cancer, here, we investigated the activation status and mechanisms of regulation of Rac1 in prostate cancer cell models. We found that activated levels of Rac1 are prominently elevated in the androgen-independent DU145 and PC3 cell lines. An exhaustive analysis of the mechanisms implicated in Rac1 hyperactivation revealed that this dysregulated signal is independent of P-Rex1 activity or other Rac-GEFs expressed in prostate cancer cells. Moreover, we made the unexpected observation that elevated Rac1-GTP levels in androgen-independent prostate cancer cells cannot be reduced by expression of a Rac-GAP, but on the other hand they are sensitive to elevations in intracellular calcium, suggesting alternative regulatory mechanisms for this small GTPase in prostate cancer models.

## 2. Results

### 2.1. Aggressive Androgen-Independent Prostate Cancer Cells Display Elevated Rac1-GTP Levels

**Figure 1:**
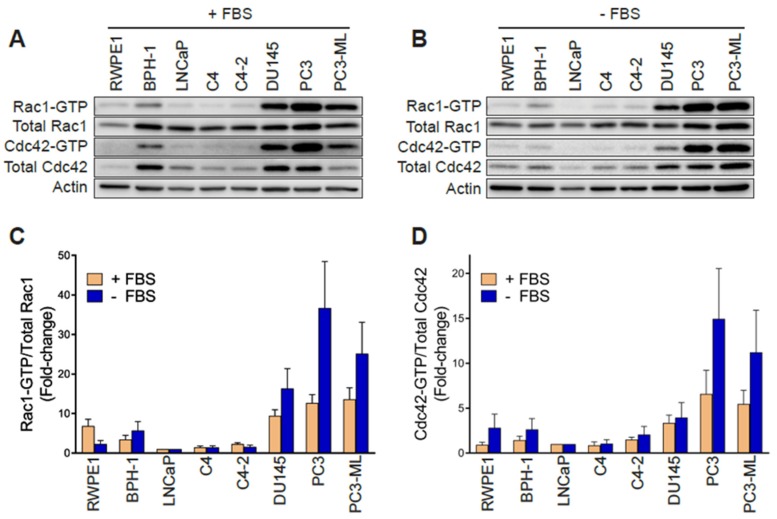

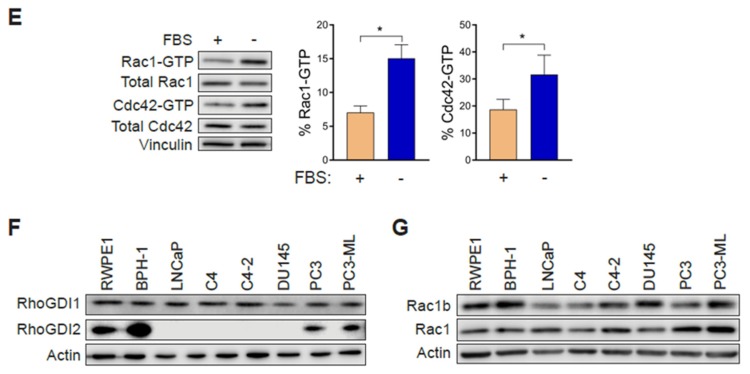
Rac1 and Cdc42 are hyperactivated in aggressive androgen-independent prostate cancer cells. (**A**) Representative Western blots of PAK-binding domain (PBD) pull-down assays showing Rac1-GTP and Cdc42-GTP in cells grown in the presence of 10% FBS. (**B**) Similar experiments were carried out after 24 h serum starvation. (**C**,**D**) Densitometric analysis of Rac1-GTP/total Rac1 and Cdc42-GTP/total Cdc42. Results normalized to LNCaP cells are expressed as the mean ± S.E. (*n* = 4–5). (**E**) Active Rac1 and Cdc42 fractions in PC3 cells growing in the presence or absence of 10% FBS. Results are expressed as the mean ± S.E. (*n* = 7) of the percentage of total Rac1 or Cdc42. * *p* < 0.05. (**F**) Representative Western blots of RhoGDI1 and RhoGDI2 expression in prostate cell lines. (**G**) Representative Western blot of Rac1b expression in prostate cancer cell lines.

In order to establish whether changes in Rac1 expression or activation occur in aggressive prostate cancer cells, we first determined the total and active (GTP-bound) Rac1 levels. For this analysis, we used non-transformed epithelial cancer cells (RWPE-1, BPH-1), androgen-dependent LNCaP cells, its derivatives C4 and C4-2, as well as aggressive androgen-independent DU145, PC3 and PC3-ML cell lines. Although no major differences in total Rac1 levels could be detected between the prostate cell lines, the assessment of Rac1-GTP levels by a PAK-binding domain (PBD) pull-down assay revealed significantly higher levels of active Rac1 in androgen-independent DU145, PC3, and PC3-ML cells relative to the other cell lines. A quantitative analysis of Rac-GTP levels normalized to total Rac1 showed ~10–15-fold elevation in active Rac1 in DU145, PC3 and PC3-ML cells compared to LNCaP cells (Figure 1A,C). A similar trend was observed for the related small GTPase Cdc42, with the highest Cdc42-GTP levels found in PC3 and PC3-ML cells (Figure 1A,D). These differences were even larger when Rac1-GTP and Cdc42-GTP levels were determined in serum-starved cells, particularly in PC3 and PC3-ML cells (~25–35-fold higher for Rac1-GTP and 10–15-fold higher for Cdc42-GTP, respectively, relative to LNCaP cells) (Figure 1B–D). This was also seen when the androgen-independent PC3 cells were compared to androgen-dependent VCaP prostate cancer cells, both in the presence and absence of FBS (Figure S1A–D). Estimation of the active fraction of these GTPases in PC3 cells revealed that 7% of Rac1 and 18% of Cdc42 were active in the presence of 10% FBS. These values were higher in serum-starved cells (15% for Rac1-GTP and 31% for Cdc42-GTP) (Figure 1E). Taking into consideration that serum and growth factors activate Rac1 and Cdc42 in most cellular models, the elevated Rac1 and Cdc42 activation after FBS removal was unanticipated.

Hyperactivation of Rho GTPases may be the consequence of mutations (e.g., Rac1^P29S^ in melanoma). To assess whether this was the case in the prostate cancer cells, we carried out Sanger sequencing. This analysis revealed no mutations in Rac1 or Cdc42 in PC3, DU145 or LNCaP cells. Likewise, no mutations were found in RhoGDI1 or RhoGDI2 in these cell lines. This agrees with genome sequence data available from the cancer cell lines encyclopedia (CCLE) (http://www.broadinstitute.org/ccle). Western blots also failed to reveal RhoGDI1 and RhoGDI2 loss of expression in the aggressive prostate cancer cell lines that could have explained Rac1 and Cdc42 hyperactivation (Figure 1F). No expression of RhoGDI3 could be detected by qPCR in any prostate cell lines used in this study. Similarly, we did not observe any appreciable correlation between the expression of the constitutively active Rac1b variant and Rac1-GTP levels in the prostate cell models (Figure 1G).

### 2.2. Elevated Rac1-GTP in Prostate Cancer Is Independent of P-Rex1

**Figure 2:**
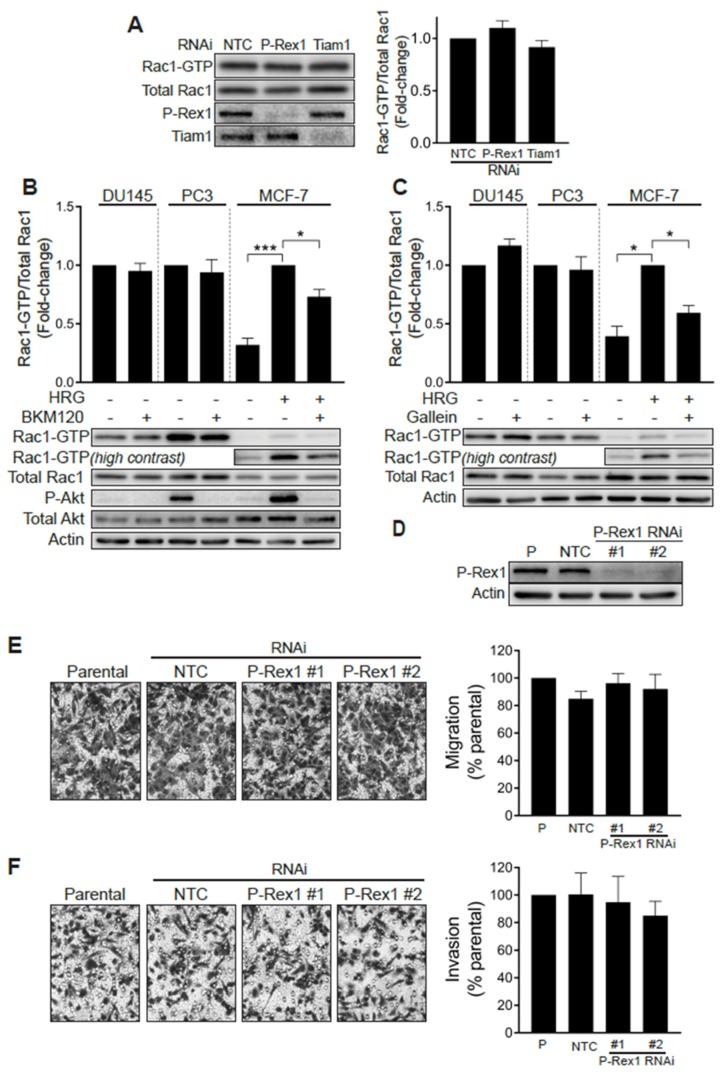
Rac1 hyperactivation in prostate cancer cells is independent of P-Rex1. (**A**) PC3 cells were transfected with P-Rex1, Tiam1 or non-target control (*NTC*) RNAi duplexes for 48 h, including a 24 h serum starvation prior to Rac1-GTP levels being determined using a PBD pull-down assay. *Left panel*, representative experiment. *Right panel*, densitometric analysis of Rac1-GTP levels normalized to total Rac1. Results, relative to NTC, are expressed as the mean ± S.E. (*n* = 6). (**B**) Effect of the PI3K inhibitor BKM120 (2 μM, 60 min) on basal Rac1-GTP levels in DU145 and PC3 prostate cells, and on HRG-stimulated (20 ng/mL, 5 min) MCF-7 breast cancer cells. (**C**) Effect of the Gβγ inhibitor gallein (10 μM, 30 min) on basal Rac1-GTP levels in DU145 and PC3 prostate cells, and on HRG-stimulated (20 ng/mL, 5 min) MCF-7 breast cancer cells. For B and C, lower panels are representative experiments. Densitometric analyses of Rac1-GTP levels normalized to total Rac1 are expressed as the mean ± S.E. (*n* = 3–4) relative to non-treated cells (PC3 and DU145) or HRG-stimulated cells (MCF-7). (**D**) Representative Western blot of P-Rex1 expression in PC3 cells subjected to P-Rex1 silencing for 48 h using two different RNAi duplexes (#1 and #2). (**E**) PC3 migration assay using a Boyden chamber without Matrigel. (**F**) PC3 invasion assay using a Boyden chamber with Matrigel. For (**E**,**F**), representative experiments are shown (*right panels*). A quantitative analysis of migration and invasion is shown in the *left panels*. Results normalized to parental cells (P) are expressed as the mean ± S.E. (*n* = 3). * *p* < 0.05; *** *p* < 0.001.

A previous study linked the PI3K- and Gβγ-regulated Rac-specific GEF P-Rex1 to metastatic phenotypes in prostate cancer. The up-regulation of P-Rex1 in PC3 cells relative to LNCaP cells or non-transformed prostate epithelial cells has been associated with Rac1 activation and migration [28]. We therefore sought to define whether P-Rex1 was responsible for the high basal Rac1-GTP levels observed in prostate cancer cell lines. To address this question, we knocked down P-Rex1 in PC3 cells with a pooled RNAi, which caused >90% reduction in P-Rex1 protein levels. Our results revealed that silencing P-Rex1 did not have any effect on Rac1-GTP levels in PC3 cells (Figure 2A). Silencing other PI3K-dependent Rac-GEFs expressed in prostate cancer cells, namely Tiam1, Vav2 or Vav3, also failed to reduce Rac1-GTP levels in PC3 cells (Figure 2A and Figure S1G).

To further validate the P-Rex1 independence of Rac1 activation in prostate cancer cells, we examined the effect of the PI3K inhibitor BKM120 and gallein, an agent that disrupts Gβγ signaling. As shown in Figure 2B,C, neither of these agents were able to reduce Rac1-GTP levels in serum-starved DU145 or PC3 cells. As a control for the inhibitors, we measured the activation of Rac1 by the ErbB3 ligand heregulin-1 (HRG) in MCF-7 breast cancer cells, a response known to be dependent on P-Rex1 activity [26]. In this case, both BMK120 and gallein significantly reduced HRG-induced Rac1 activation. The efficacy of the PI3K inhibitor was also confirmed by its ability to reduce phospho-Akt levels in PC3 and in HRG-stimulated MCF-7 cells. BKM120 and gallein also failed to reduce Cdc42-GTP levels in DU145 and PC3 cells (Figure S1E,F). These results suggest that Rac1 and Cdc42 hyperactivation in androgen-independent prostate cancer cells does not involve aberrant upstream signaling mediated by PI3K/Gβγ -dependent exchange factors, such as P-Rex1.

We then sought to establish whether P-Rex1 plays a role in prostate cancer cell migration and invasion. Consistent with the lack of involvement for P-Rex1 in regulating Rac1 activation in PC3 cells, silencing the expression of this Rac-GEF failed to impair cell motility, as determined with a Boyden chamber assay (Figure 2D,E). Likewise, the ability of PC3 cells to invade through Matrigel was not affected by P-Rex1 RNAi mediated depletion (Figure 2F). Taken together, these results confirmed that P-Rex1 does not contribute to maintaining high Rac1-GTP levels and is dispensable for migration or invasion of androgen-independent prostate cancer cells.

**Figure 3:**
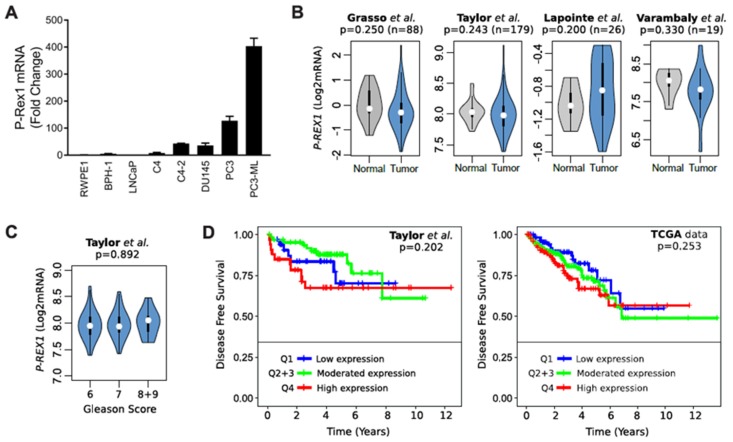
Expression of P-Rex1 in prostate cancer. (**A**) mRNA expression level of P-Rex1 in the indicated prostate cancer cell lines was assessed by RT-qPCR. (**B**) Violin plots showing the status of P-Rex1 mRNA expression in prostate cancer tumor and normal tissues. (**C**) Violin plots showing P-Rex1 mRNA expression relative to Gleason score. (**D**) Kaplan–Maier curves showing the disease-free survival of patient groups selected according to the *PREX1* quartile expression. Analysis was performed using publicly available datasets from (**B**) Grasso et al. [31], Lapointe et al. [32], and Varambaly et al. [33]. (**B**,**D**) Taylor et al. [34] and (**D**) TCGA.

Analysis of P-Rex1 mRNA expression showed a prominent up-regulation in androgen-independent prostate cancer cells, most prominently in the bone metastatic PC3-ML variant (Figure 3A). However, analysis of publicly available databases did not show appreciable changes of P-Rex1 expression in normal vs. human prostate tumors (Figure 3B). In addition, no significant changes in P-Rex1 expression were detected based on Gleason Score (Figure 3C). There were also no differences in disease free survival (DFS) based on P-Rex1 mRNA expression (Figure 3D). Altogether, this analysis suggests that P-Rex1 may not have a significant role in human prostate cancer progression, unlike that described in other cancer types.

### 2.3. Elevated Rac1 Activation in Androgen-Independent Prostate Cancer Cells Is Insensitive to the Rac-GAP β2-Chimaerin

**Figure 4:**
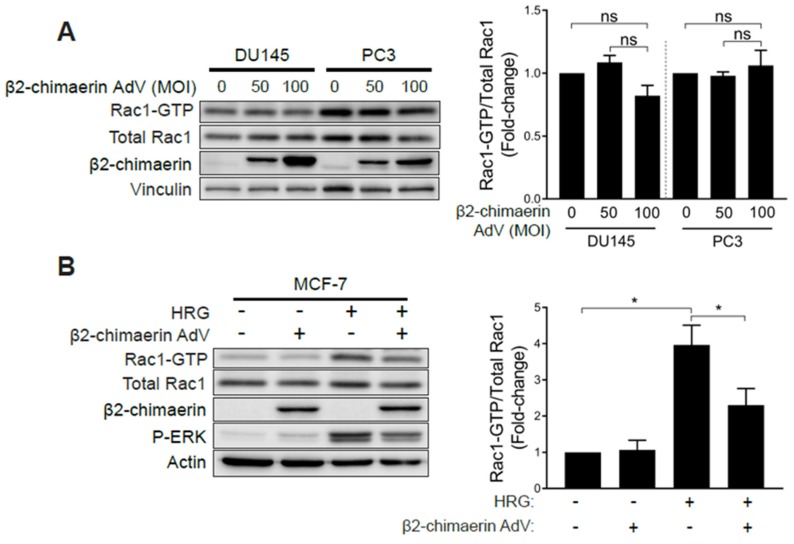
Elevated Rac1 activation in androgen-independent prostate cancer cells is insensitive to the Rac-GAP β2-chimaerin. (**A**) DU145 and PC3 cells were infected with increasing MOIs (0–100 pfu/cell) of a β2-chimaerin AdV. After 16 h, Rac1-GTP levels were determined using a PBD pull-down assay. (**B**) Similar experiments were carried out in MCF-7 cells stimulated with HRG (20 ng/mL, 5 min). *Left panels*, representative experiments. *Right panels*, densitometric analyses of Rac1-GTP/total Rac1. Results (normalized to MOI = 0 pfu/cell) are expressed as the mean ± S.E. (*n* = 3–4). *, *p* < 0.05; n.s., not significant.

One explanation for the observed hyperactivation of Rac1 in androgen-independent prostate cancer cells is that it could be the consequence of deficient expression of GAPs responsible for Rac1 inactivation. To address this issue, we overexpressed the Rac-specific GAP β2-chimaerin in prostate cancer cells using an adenoviral approach [35]. β2-chimaerin was chosen based on its robust ability to deactivate Rac1 in other cell types [35,36,37]. To our surprise, ectopic expression of β2-chimaerin in PC3 or DU145 cells did not significantly affect Rac-GTP levels (Figure 4A). On the other hand, β2-chimaerin abrogated Rac1 activation by HRG in MCF-7 breast cancer cells, as we have reported in a previous study [38] (Figure 4B). As a control, ectopically expressed β2-chimaerin could be pulled down from both PC3 and MCF-7 cell extracts using a constitutively active Rac1^Q61L^ bait (Figure S2A), as expected based on the preferential ability of Rac-GAPs to bind activated Rac1. Therefore, elevated Rac1-GTP levels in androgen-independent prostate cancer cells are insensitive to Rac-GAP inactivation. The small GTPase RhoA has previously been shown to negatively regulate Rac1 via the activation of a Rac-GAP in other systems [39]. However, treatment of PC3 cells with the small molecule Rho Activator II [40] had no effect on the level of Rac1 activation, therefore demonstrating that the pool of hyperactive Rac1 in these prostate cancer cells is also insensitive to negative regulation by RhoA (Figure S2B).

### 2.4. Silencing Rac-GEFs in PC3 Cells Did Not Impact Rac1-GTP Levels

The insensitivity of Rac1-GTP levels to Rac-GAP overexpression could be due to the activity of a particularly dominant Rac-GEF driving Rac1 hyperactivation. To address this issue, we decided to use an RNAi approach to individually silence Rac-GEFs expressed in aggressive prostate cancer cells. First, we examined the expression of Rac-GEFs in PC3 cells using qPCR (Figure S3A). The analysis showed that 34 GEFs (23 dbl-like GEFs and 11 DOCK GEFs) could be detected at the mRNA level. We were unable to detect the expression of RasGRF1, ARHGEF15, ECT2L, VAV1, MCF2, FGD5, KALRN, RasGRF2 and ARHGEF19.

**Figure 5:**
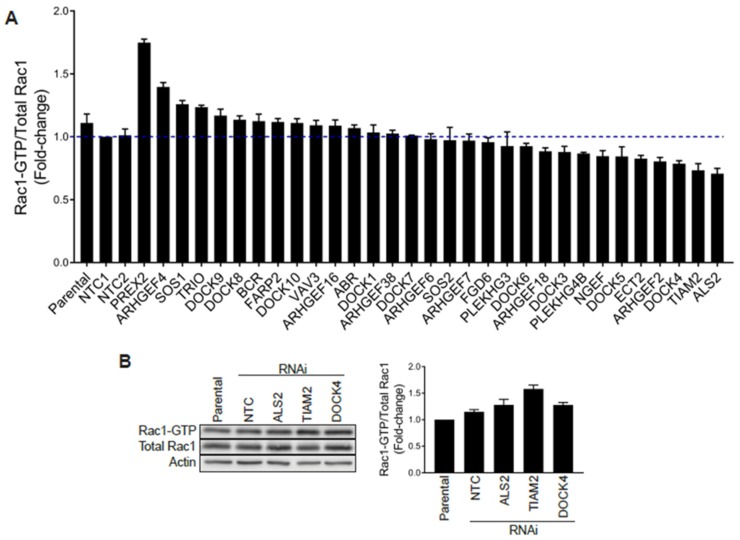
G-LISA screen of Rac-GEFs. (**A**) PC3 cells with stable depletion of indicated Rac-GEFs were serum starved and Rac1-GTP levels analyzed using a G-LISA assay screen. Rac1-GTP levels, normalized to total Rac1 assessed by Western blot and expressed relative to NTC1, are expressed as the mean of three replicates ± S.D. (**B**) PC3 cells were transfected with siRNA duplexes targeting ALS2, TIAM2, DOCK4 or a non-target control (*NTC*), and Rac1-GTP levels measured using a PBD assay after 48 h. *Left panel*, representative experiment. *Right panel*, densitometric analysis of Rac1-GTP/total Rac1. Results, relative to parental cells, are expressed as the mean ± S.E. (*n* = 3).

Stable GEF-depleted PC3 cell lines were then generated upon infection with pre-designed shRNA lentiviruses, followed by puromycin selection. For each GEF we used 2–5 different shRNA lentiviruses, totaling 110 puromycin-resistant stable cell lines for a total of 34 GEFs. We then selected for each GEF the stable cell line with the greatest level of silencing, as determined by qPCR (Figure S3B), and examined their Rac1-GTP levels using a G-LISA approach. Stable depletion of the GEFs DOCK2, DOCK6 and DOCK11 by any of the viruses used was deemed insufficient (<50%) and these were therefore not included in the analysis. As shown in Figure 5A, none of the GEF-deficient cell lines showed major reductions in Rac1-GTP levels (normalized to total Rac1) relative to parental cells or cells subject to non-target control shRNA, other than a relatively minor effect for DOCK4-, TIAM2- and ALS2-depleted PC3 cell lines. However, a similar analysis carried out using siRNA duplexes for DOCK4, TIAM2 or ALS2 revealed no change in Rac1-GTP levels (Figure 5B and Figure S4A), suggesting that the effect observed with shRNAs for these GEFs was most likely an off-target effect. Of note, silencing the expression of these GEFs did not cause a reduction in the Cdc42-GTP levels (Figure S4B). An increase in Rac-GTP levels in the G-LISA screening was observed upon silencing P-Rex2 and ARHGEF4. However, this effect was most likely a consequence of a reduction in total Rac1 levels that translated into an elevated Rac1-GTP/total Rac1 ratio. Based on these results, we concluded that no single Rac-GEF tested is responsible for Rac1 hyperactivation in prostate cancer.

### 2.5. Identification of Rac1 Interacting Partners in Prostate Cancer Cells Using Mass Spectrometry

In a further effort to elucidate the mechanisms responsible for mediating Rac1 hyperactivation in androgen-independent prostate cancer cells, we performed mass spectrometry with the goal of identifying Rac1 binding partners. For this analysis, we used a GST-tagged G15A Rac1 mutant (nucleotide-free) as a bait, an approach that is used to pull-down associated GEFs [41]. Mass spectrometry analysis identified 301 potential Rac1 binding partners in PC3 cell lysates (Table S1). Notably, none of these Rac1 associated proteins were known Rac-GEFs, further reinforcing the lack of involvement of these nucleotide-exchange factors. The analysis revealed a number of proteins that based on the literature could be potentially involved in regulating Rac1 signaling. The most prominent candidates were SmgGDS (a Rac1 interacting protein that contributes to its activation by Rac-GEFs [42]) and Grb2 (shown to facilitate the activation of Rac1 by a GEF [43]). Therefore, we used a silencing approach to knockdown SmgGDS and Grb2 expression, followed by the determination of Rac-1-GTP levels. These experiments revealed that neither of these interacting proteins were implicated in maintaining elevated Rac1-GTP levels in PC3 cells (Figure S5A,B).

### 2.6. Rac1 Hyperactivation in PC3 Cells Is Reduced in Response to Serum and Elevations in Intracellular Calcium

**Figure 6:**
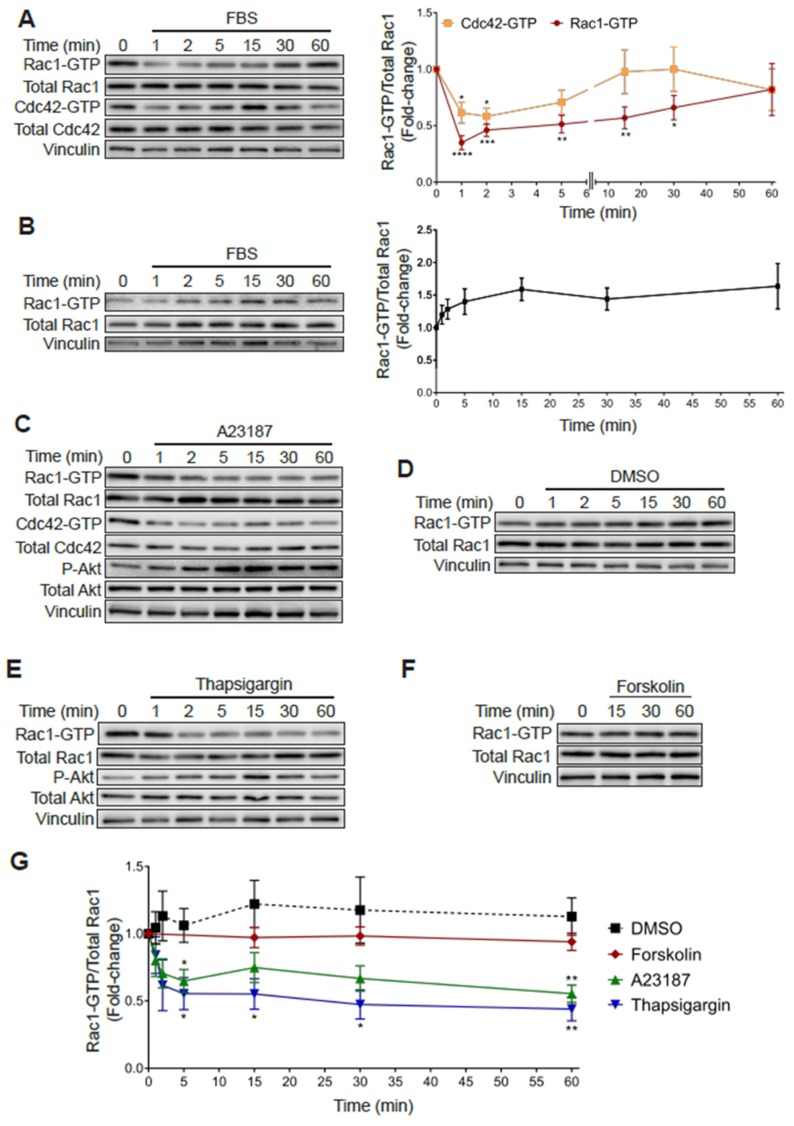
Increased Rac1-GTP levels in PC3 cells are sensitive to elevations in intracellular calcium. (**A**) Serum starved PC3 cells were stimulated with FBS (10%) for the indicated time and a PBD pull-down assay was performed to determine Rac1-GTP and Cdc42-GTP levels. (**B**) Similar experiments were carried out in LNCaP cells. For (**A**,**B**) *Left panels*, representative experiments. *Right panels*, densitometric analysis of Rac1-GTP levels normalized to total Rac1, and Cdc42-GTP levels normalized to total Cdc42. (**C**–**F**) Serum starved PC3 cells were treated with DMSO (0.1%), A23187 (2 μM), thapsigargin (1 μM) or forskolin (1 μM) for the indicated times, and PBD pull assays were performed to determine Rac1-GTP and Cdc42-GTP levels. (**C**–**F**) Representative experiments are shown for (**C**) DMSO, (**D**) A23187, (**E**) thapsigargin and (**F**) forskolin treatment. (**G**) Densitometric analysis of Rac1-GTP levels normalized to total Rac1. Results, relative to the unstimulated time point, are expressed as the mean ± S.E. (*n* = 3–7). * *p* < 0.05; ** *p* < 0.01; *** *p* < 0.001; **** *p* < 0.0001.

As shown in Figure 1, subjecting PC3 cells to 24 h serum starvation resulted in an increase in Rac1-GTP and Cdc42-GTP levels relative to cells growing in medium with FBS. Notably, addition of 10% FBS to serum starved PC3 cells led to a rapid decrease in Rac1-GTP and Cdc42-GTP levels. Indeed, 65% and 40% reductions in Rac-GTP and Cdc42-GTP levels, respectively, were observed at 1 min after FBS addition, which progressively returned to the high basal levels after 20–60 min (Figure 6A). In contrast, androgen-dependent LNCaP prostate cancer cells, which have a low basal Rac1-GTP level, displayed a moderate increase in Rac1-GTP in response to serum, peaking at 15 min (Figure 6B).

In an effort to identify the mechanisms responsible for this effect, we examined candidate second messengers capable of affecting Rac1 and Cdc42 activation. Considering that multiple extracellular cues lead to elevations in calcium levels, we examined the effect of the calcium ionophore A23187. Notably, treating PC3 cells with this ionophore led to a rapid and sustained reduction in Rac1-GTP and Cdc42-GTP that was not observed with the vehicle DMSO (Figure 6C,D). As a second approach to evaluate the role of intracellular calcium in small GTPase deactivation, we evaluated the effect of thapsigargin, a sarcoplasmic/endoplasmic reticulum Ca^2+^-ATPase (SERCA) pump inhibitor that promotes a significant elevation in intracellular calcium [44]. Like the calcium ionophore, thapsigargin also reduced Rac1-GTP levels in PC3 cells (Figure 6E). In contrast, stimulation of cAMP production with forskolin did not cause a significant effect (Figure 6F). A quantitative analysis of these effects is depicted in Figure 6G. Therefore, this sensitivity to thapsigargin and the ionophore A23187 demonstrates a role for calcium-signaling events in mediating the rapid inactivation of Rac1-GTP and Cdc42-GTP in PC3 cells.

## 3. Discussion

In this study, we investigated the regulation of Rac1 in prostate cancer cellular models. The main findings of our study are three-fold. First, we demonstrated that aggressive androgen-independent prostate cancer cell lines have markedly increased levels of activated Rac1, an effect also observed for the related G-protein Cdc42. Second, we demonstrated that the Rac-GEF P-Rex1, which has been previously reported as a key Rac1 activator in prostate cancer models, is dispensable for conferring Rac1 hyperactivation. Along this line, no other Rac1-GEF or mutations in Rac1 itself or in RhoGDIs could explain the elevation of Rac1-GTP in androgen-independent prostate cancer cells. Third, the elevated Rac1-GTP levels in these cells are not subject to the typical regulatory mechanisms described for Rac GTPases in other contexts, as judged by the unexpected insensitivity to a Rac-GAP and its deactivation via calcium-mediated mechanisms. This raises the possibility that yet unidentified regulatory mechanisms take place in the control of Rac1 activation in prostate cancer cells.

Contrarily to a previous report implicating P-Rex1 up-regulation in the maintenance of Rac1 hyperactivation [28] in androgen-independent prostate cancer cells, our study shows no effect of P-Rex1 silencing on Rac1-GTP levels in this model. Consistent with this, P-Rex1 was also found to be dispensable for the migration or invasion of PC3 cells. Although Qin et al. [28] reported a reduction in Rac1-GTP levels in PC3 cells by delivery of a single P-Rex1 siRNA duplex. In our hands, the delivery of multiple RNAi duplexes failed to have any effect. The observed differences may relate to the different experimental approaches in each case. In support of our finding, PI3K and Gβγ inhibitors, which abrogate P-Rex1 activation, had no effect on Rac1-GTP levels in androgen-independent prostate cancer cells. It is important to note, however, that the early study [28] reported significant reductions in CXCR4- and EGFR-mediated PC3 motility, which fits with the proposed mechanisms of P-Rex1 activation by receptor stimulation. In this regard, up-regulated P-Rex1 levels in luminal breast cancer cells do not cause marked changes in basal Rac1-GTP but rather sensitize Rac1 activation to the stimulation of tyrosine-kinase receptors and GPCRs [26,30].

Previous work also identified an up-regulation of P-Rex1 mRNA in PC3 cells compared to less aggressive prostate cancer cells such as LNCaP [28], similar to our observation in PC3 and PC3-ML cells. However, analysis of publicly available human gene expression databases revealed no correlation of P-Rex1 expression with cancer status, Gleason score or disease-free survival. Therefore, the observed up-regulation of P-Rex1 in PC3 cells does not seem to be representative of a general occurring effect in human prostate cancer. These results argue that P-Rex1 may not represent a promising therapeutic target or biomarker of prostate cancer progression as otherwise suggested in luminal breast cancer and melanoma [27,30,45].

Our screening of Rac-GEF-depleted PC3 cell lines was unable to identify a single exchange factor responsible for Rac1 hyperactivation. There are several possible scenarios explaining this result. One is that multiple GEFs are required for maintaining Rac1-GTP and Cdc42-GTP in these cells, and thus silencing single GEFs is insufficient to cause an effect. We cannot rule out that a Rac-GEF not included in our screen could be involved, albeit a less likely possibility because our analysis encompasses the majority of known Rac-GEFs [3]. However, it is quite remarkable that no Dbl or DOCK family GEFs were identified in our proteomics analysis using a nucleotide free Rac1 bait, which is designed to pull-down Rac1 associated GEFs. Interestingly, the proteomics detected the atypical GEF SmgGDS, a protein reported to interact with Rac1 that indirectly contributes to Rac1 regulation [42,46]; however, this atypical GEF was found to be dispensable for Rac1 hyperactivation in androgen-independent prostate cancer cells. Another possible alternative explanation for the lack of dependency on any of the tested GEFs is that a non-GEF protein promotes or is required for maintaining the active Rac1 and Cdc42 status. For example, a protein could stay bound to the GTPases to prevent their inactivation by GAPs. This would indeed fit with our observation that β2-chimaerin overexpression failed to reduce Rac1-GTP levels in both DU145 and PC3 prostate cancer cells while still achieving a reduction in MCF-7 breast cancer cells. One such candidate is IQGAP1, a scaffold protein that binds to Rac1-GTP and Cdc42-GTP and contributes to the organization of a multimolecular complex involved in regulation of the actin cytoskeleton [47]. Although IQGAP1 was not identified in our proteomics screen, suppressing its expression by RNAi did not affect Rac1 or Cdc42 hyperactivation in PC3 cells (Figure S5C). Another scenario is that the Rac1-GTP pool in androgen-independent prostate cancer cells is located in a cellular compartment that is not accessible to regulatory proteins. In this regard, Rac1 could be localized to distinct organelles where it has specialized functions. However, in the absence of suitable tools to visualize the intracellular localization of endogenous Rac1-GTP we could not address this question.

Finally, our results revealed an unexpected mechanism of Rac1 and Cdc42 deactivation in aggressive androgen-independent prostate cancer cells. The observation that PC3 cells display elevated Rac1 and Cdc42 activation in response to serum starvation suggests the presence of a factor in the serum that was suppressing the activation of these GTPases. This was confirmed by the effect of FBS stimulation on starved cells, which produced a rapid reduction in the levels of Rac1-GTP and Cdc42-GTP. This loss of active G-protein could be mimicked by pharmacological agents that increase intracellular calcium levels, either a calcium ionophore or a SERCA pump inhibitor, but not by a cAMP-generating agent. These results were unforeseen since the stimulation of cells with FBS or growth factors is generally expected to increase Rac1 activation, as we observed in LNCaP cells and as previously reported in other cell types [48,49]. Furthermore, calcium-signaling events in other cell types and contexts has been shown to indirectly regulate the activation of Rac1 [50,51,52]. It is therefore remarkable that a rise in intracellular calcium results in the deactivation of Rac1 and Cdc42 in PC3 cells and may indicate the presence of a potent calcium-stimulated GAP or the calcium dependent dissociation of a binding partner that otherwise sustains hyperactivation of these GTPases. Such a mechanism may be quite restricted by the localization of Rac1 and Cdc42 in androgen-independent prostate cancer cells.

In summary, our work identified Rac1 and Cdc42 hyperactivation in aggressive androgen-insensitive DU145 and PC3 prostate cancer cell lines. These proteins and their regulators are known to play important roles in the progression of cancer and are therefore of interest as potential therapeutic targets [3]. We have also demonstrated that targeting P-Rex1, a previously identified therapeutic target candidate, is unlikely to have an impact on Rac1 activation status, migration or invasion in prostate cancer cells. Considering the essential roles Rac1 and related GTPases proteins have in metastasis, deciphering the molecular mechanisms behind their unusual regulation in prostate cancer is of utmost importance.

## 4. Materials and Methods

### 4.1. Cell Lines, Cell Culture, and Reagents

Human prostate cancer cell lines (LNCaP, LNCaP-C4, LNCaP-C4-2, DU145, PC3) and normal prostate epithelial cells (RWPE1) were obtained from ATCC (Rockville, MD, USA). PC3-ML cells were obtained from Dr. Alessandro Fatatis (Drexel University, Philadelphia, PA, USA), as previously described [53]. BPH-1 cells were a kind gift from Dr. Cecilia Caino (University of Colorado, Denver, CO, USA). VCaP cells were kindly provided by Dr. Trevor M. Penning (University of Pennsylvania, Philadelphia, PA, USA). Heregulin-β1 was purchased from R&D Systems (Minneapolis, MN, USA) and FBS from Hyclone. BKM120 was obtained from Santa Cruz Biotechnology (Santa Cruz, CA, USA). Gallein was purchased from Tocris Bioscience (Bristol, UK). Forskolin, A123187 and thapsigargin were obtained from Sigma-Aldrich (St. Louis, MO, USA). Rho Activator II was purchased from Cytoskeleton (Denver, CO, USA).

### 4.2. RNA Interference (RNAi)

Transfection of siRNA duplexes was performed with Lipofectamine RNAiMax following the manufacturer’s protocol. The following ON-TARGET RNAi duplexes were obtained from Dharmacon (Lafayette, CO, USA): P-Rex1 (L-010063-01, J-010063-10, J-010063-11), Vav2 (L-005199-00), Vav3 (L-010178-00), Als2 (L-014168-00), DOCK4 (L-017968-01), Tiam2 (L-008434-00), SmgGDS (J-015881-05), Grb2 (J-019220-07), IQGAP1 (L-004694-00) and non-target control (LD-001810-10).

### 4.3. Generation of Stable GEF-Depleted Cell Lines

Lentiviral constructs targeting Rac-GEFs and pLKO non-targeting control shRNA constructs were obtained from Sigma-Aldrich. pLKO.1 shRNAs for human ABR (TRCN0000047948), ALS2 (TRCN0000047805), ARHGEF2 (TRCN0000003175), ARHGEF4 (TRCN0000047559), ARHGEF6 (TRCN0000007403), ARHGEF7 (TRCN0000047596), ARHGEF16 (TRCN0000047505), ARHGEF18 (TRCN0000047534), ARHGEF19 (TRCN0000048239), ARHGEF38 (TRCN0000106214), BCR (TRCN0000000789), DOCK1 (TRCN0000029077), DOCK3 (TRCN0000122974), DOCK4 (TRCN0000039730), DOCK5 (TRCN0000113802), DOCK7 (TRCN0000129042), DOCK8 TRCN0000122957), DOCK9 (TRCN0000141047), DOCK10 (TRCN0000122950), ECT2 (TRCN0000047684), FARP2 (TRCN0000047259), FGD5 (TRCN0000048244), FGD6 (TRCN0000047268), KALRN (TRCN0000001430), NGEF (TRCN0000047657), PLEKHG3 (TRCN0000048681), PLEKHG4B (TRCN0000156705), PREX2 (TRCN0000157338), SOS1 (TRCN0000048143), SOS2 (TRCN0000048153), TIAM2 (TRCN0000107211), TRIO (TRCN0000000871), and VAV3 (TRCN0000047699) were obtained from Open Biosystems (Huntsville, AL, USA). Lentiviruses were produced by the Lenti-shRNA Core Facility (UNC-Chapel Hill, NC, USA). Cells were infected with lentivirus particles overnight before selection the following day with puromycin (1 μg/mL).

### 4.4. Rac1-GTP/Cdc42-GTP Pull-Down (PBD) Assays

The pull-down assay for determining Rac1-GTP and Cdc42-GTP levels was performed as previously described and is based on the binding of the active G-protein to the PAK-binding domain (PBD) [36,54]. For some experiments, cells were serum starved for 24 h before the assay.

### 4.5. G-LISA Assay for Rac1-GTP

The colorimetric G-LISA kit (BK128, Cytoskeleton, Denver, CO, USA) was used to measure Rac1-GTP levels in the stable GEF-depleted cell lines. Briefly, cells were grown for 24 h, followed by 24 h serum starvation immediately prior to the assay. Cells were lysed with kit lysis buffer, lysates clarified and then normalized for total protein. G-LISA was performed following the manufacturer’s protocol. The G-LISA signal was normalized for total Rac1 levels that were assessed by Western blot. Experiments were done in triplicate.

### 4.6. Adenoviral Expression of β2-Chimaerin

Ectopic expression of β2-chimaerin was achieved with an adenoviral approach, as previously described [37,38]. The stimulation of cells, when required, was achieved with the addition of 10× stimulant to the cells in starvation media. To simultaneously analyze the activation of Rac1 and Cdc42, post-clarification lysates were divided onto separate beads for the pulldown.

### 4.7. Western Blot Analysis

Western blot was performed as previously described [55]. The following antibodies were used at 1:1000 dilution: anti-Rac1 clone 23A8 (Cat. # 05-389), anti-Rac1b (Cat. # 09-271) and anti-P-Rex1 (Cat. #MABC178) from EMD Millipore (Burlington, MA, USA); anti-Cdc42 clone 11A11 (Cat. # 2466), anti-phospho-AKT (Cat. # 9271), anti-AKT (Cat. # 4691), anti-phospho-ERK (Cat. # 9101), and anti-RhoGDI1 (Cat # 2564) from Cell Signaling Technologies (Danvers, MA, USA); anti-Tiam1 (Cat. # AF5038) from R&D Systems (Minneapolis, MN, USA); anti-β2-chimaerin (Cat. # SAB4200059) from Sigma-Aldrich (St. Louis, MO, USA); anti Ly-GDI (D7) (sc-271108) from Santa Cruz Biotechnology (Santa Cruz, CA, USA). The following antibodies, obtained from Sigma-Aldrich, were used at 1:5000 dilution: anti-actin (Cat. # A544) and anti-vinculin (Cat. # V9131). The following secondary antibodies were used at 1:3000 dilution: goat anti-mouse (Cat. # 172-1011), goat anti-rabbit (Cat. # 170-6515), and goat anti-sheep (Cat. # 172-1017) from BioRad (Hercules, CA, USA); and goat anti-rat (Cat. # 112-035-062) from Jackson ImmunoResearch (West Grove, PA, USA). Bands were visualized using enhanced chemiluminescence (ECL) with the LI-COR Odyssey Fc dual mode imaging system.

### 4.8. Analysis of mRNA Expression by qPCR

mRNA expression levels were analyzed using reverse transcription qPCR. Briefly, total RNA was extracted using the RNeasy kit (QIAGEN, Valencia, CA, USA) and cDNA produced using the Taqman reverse transcription reagent (N8080234, Applied Biosystems, Waltham, MA, USA). qPCR amplifications were performed using a QuantStudio 3 detection system using carboxyfluorescein (FAM) tagged probes (ThermoFisher, Waltham, MA, USA). The FAM signal was normalized to endogenous β2-microglobulin housekeeping gene and fold-change calculated from comparative Ct analysis.

### 4.9. Migration and Invasion Assays

Migration assays were performed with a Boyden chamber, using membranes with 12 μm pores and 5% FBS in the lower chamber as a chemoattractant. Invasion assays were performed in the same way but after coating of the upper side of the membrane with 0.5 mg/mL Matrigel. Experiments were carried out 48 h after transfection of RNAi duplexes, including a 24 h serum starvation prior to the assay, which was performed as previously described [53].

### 4.10. In silico Analysis of P-REX1 mRNA Expression among Normal and Prostate Cancer Tissues

Processed *P-REX1* mRNA expression levels (Log2 transformed) and clinicopathological data from five independent prostate cancer studies were obtained from the CancerTools resource (http://genomics.cicbiogune.es/CANCERTOOL/) [56]. Univariate and survival analysis were performed with R software.

### 4.11. Statistical Analysis

Statistical analysis was done with student’s *t*-test or ANOVA using GraphPad Prism 3.0. A *p*-value <0.05 was considered statistically significant. Statistical significance of Kaplan–Maier curves was tested with the Mantel–Cox test.

## 5. Conclusions

The present study demonstrates that Rac1 hyperactivation occurs in aggressive androgen-independent prostate cancer, and that this is independent of P-Rex1 Rac-GEF activity. Furthermore, this Rac1 hyperactivation was found to be insensitive to overexpression of the Rac-GAP β2-chimaerin. Our study also determined that increases in intracellular calcium lead to a rapid decrease in both Rac1-GTP and Cdc42-GTP levels.

## Supplementary Materials

The following are available online at https://www.mdpi.com/2072-6694/12/2/480/s1, Figure S1: Rac1-GTP and Cdc42-GTP levels in the androgen dependent VCaP cells and the effect of PI3K and Gβγ inhibition and Vav2/3 silencing in DU145 and PC3 cells, Figure S2: Association of β2-chimaerin expressed in PC3 and MCF-7 cells with a constitutively active Rac1^Q61L^ mutant and insensitivity of Rac1-GTP in PC3 cells to Rho activation, Figure S3: Expression of Rac-GEFs in PC3 cells, Figure S4: Silencing ALS2, TIAM2 or DOCK4 does not affect Cdc42-GTP levels, Figure S5: SmgGDS, Grb2 and IQGAP1 silencing have no effect on Rac1-GTP levels, Table S1: Proteomics of Rac1G15A binding proteins.
