# Supplementary material for: P-REX1-Independent, Calcium-Dependent RAC1 Hyperactivation in Prostate Cancer
Source: Cancers (Basel). 2020 Feb 19;12(2):480. doi: 10.3390/cancers12020480 (PMC7072377; doi:10.3390/cancers12020480)
Supplement: Supplementary file 1 [file cancers-12-00480-s001.zip › cancers-688668-SuppMaterials-final check/supplementary-final check.pdf]

cancers

MDPI

Supplementary Materials

P-REX1-Independent, Calcium-Dependent RAC1 Hyperactivation in Prostate Cancer

Martin J. Baker, Martín C. Abba, Rafael Garcia-Mata and Marcelo G. Kazanietz

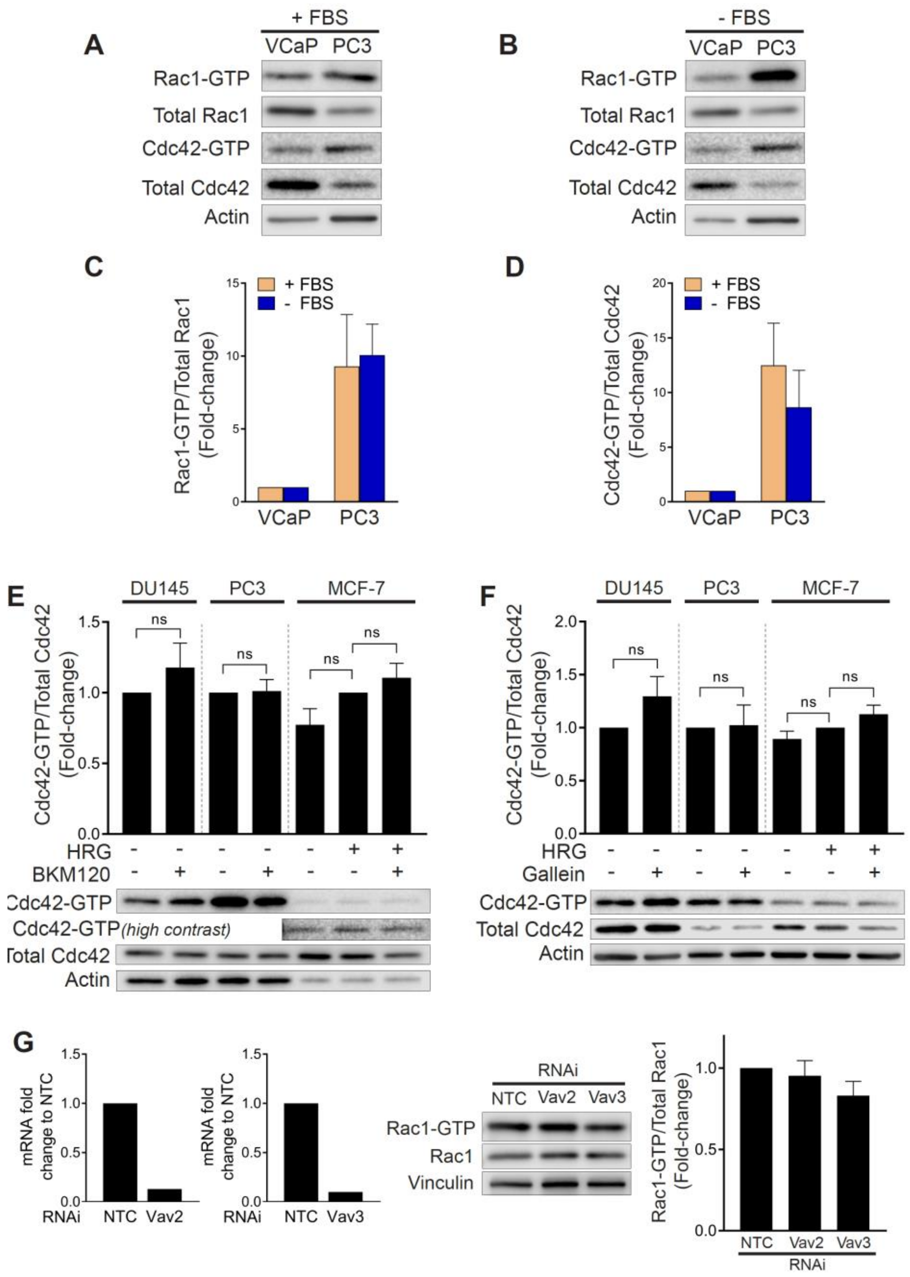

Figure S1. Rac1-GTP and Cdc42-GTP levels in the androgen dependent VCaP cells and the effect of PI3K and Gβγ inhibition and Vav2/3 silencing in DU145 and PC3 cells (**A**) Representative Western blots of PBD pull-down assays showing Rac1-GTP and Cdc42-GTP in VCaP and PC3 cells growing in the presence of 10% FBS. (**B**) Similar experiments were carried out after 24 h serum starvation. (**C–D**) Densitometric analysis of Rac1-GTP/total Rac1 and Cdc42-GTP/total Cdc42. Results normalized to VCaP cells are expressed as the mean ± S.E. (*n* = 3). (**E**) Effect of the PI3K inhibitor BKM120 (2 µM, 60 min) on basal Cdc42-GTP levels in DU145 and PC3 prostate cells, and on HRG-stimulated (20 ng/mL,

5 min) MCF-7 breast cancer cells. (**F**) Effect of the Gβγ inhibitor gallein (10 μM, 30 min) on basal Cdc42-GTP levels in DU145 and PC3 prostate cells, and on HRG-stimulated (20 ng/mL, 5 min) MCF-7 breast cancer cells. For E and F, *lower panels* are representative experiments, and *upper panels,* densitometric analysis of Cdc42-GTP levels normalized to total Cdc42. Results are expressed as the mean ± S.E. (n = 4–6) relative to non-treated cells (PC3 and DU145) or HRG-stimulated cells (MCF-7). (**G**) PC3 cells were transfected with Vav2, Vav3 or non-target control (*NTC*) RNAi duplexes, and the Rac1-GTP levels determined using a PBD pull-down assay after 48 h. *Left panel*, representative mRNA fold-change after RNAi. *Middle panel*, representative Rac1-GTP pulldown experiment. *Right panel*, densitometric analysis of Rac1-GTP levels normalized to total Rac1. Results, relative to NTC, are expressed as the mean ± S.E. (n = 3).

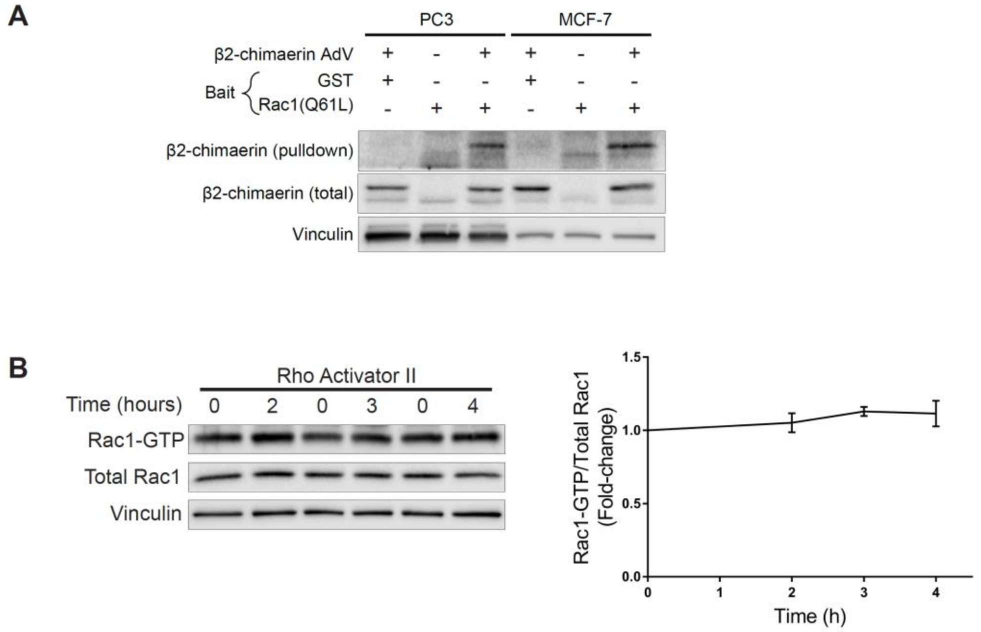

Figure S2. Association of β2-chimaerin expressed in PC3 and MCF-7 cells with a constitutively active Rac1^{Q61L} mutant and insensitivity of Rac1-GTP in PC3 cells to Rho activation. (**A**) PC3 and MCF7 cells were infected with β2-chimaerin AdV (100 pfu/cell). After 16 h, cells were lysed and recombinant GST-fused Rac1^{Q61L} bait was used to pull-down β2-chimaerin. A representative experiment is shown. (**B**) PC3 cells were treated for the indicated times with 1 μg/mL Rho Activator II and Rac1-GTP levels determined using the PBD pulldown assay. *Left panel*, representative experiment. *Right panel*, densitometric analysis of Rac1-GTP levels normalized to total Rac1 levels. Results are expressed as the mean ± S.E. (n = 3) relative to non-treated cells.

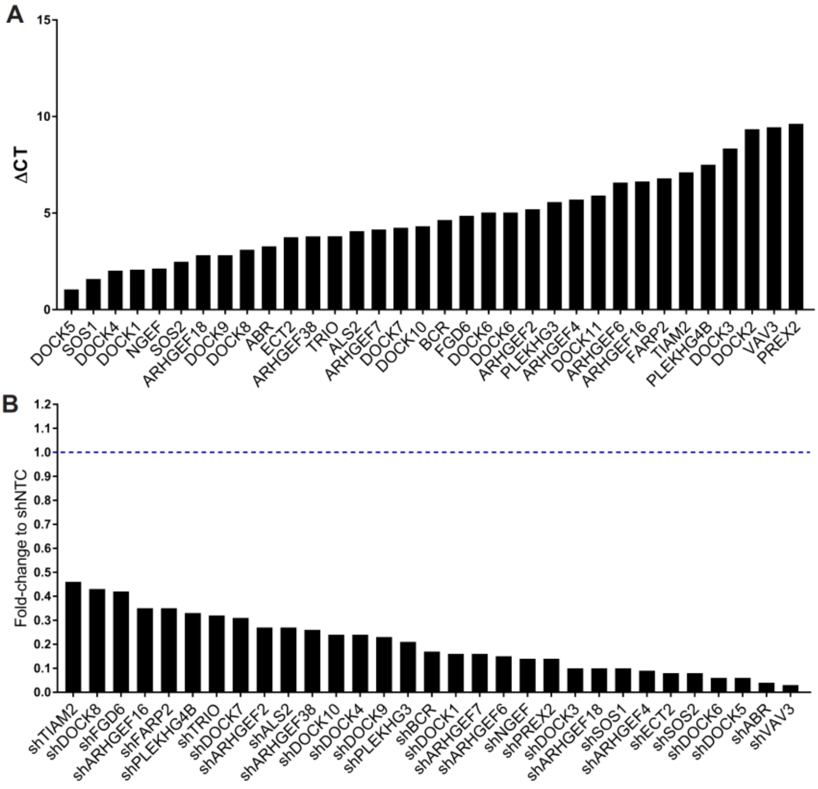

Figure S3. Expression of Rac-GEFs in PC3 cells. (**A**) Expression of Rac-GEFs in the stable non-target control (shNTC1) PC3 cell line by qPCR. Results are expressed as ΔCT, using β2-microglobulin as a housekeeping gene. (**B**) mRNA expression levels of the indicated Rac-GEF in each of the stable depleted cell lines are displayed as fold-change relative to the shNT1 cell line. The depletion of FGD5 resulted in undetectable levels of mRNA in the corresponding stably depleted cell line.

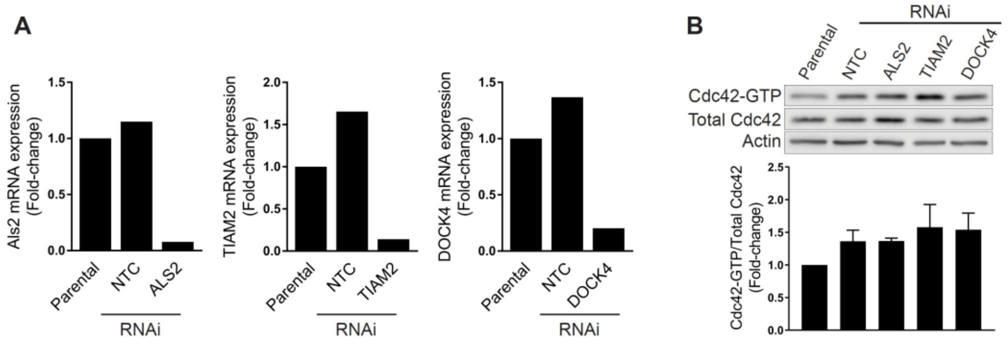

Figure S4. Silencing ALS2, TIAM2 or DOCK4 does not affect Cdc42-GTP levels. (**A**) Representative mRNA expression fold-change for ALS2, TIAM2 and DOCK4 48 h after transfection of RNAi duplexes. (**B**) PC3 cells were transfected with RNAi targeting ALS2, TIAM2, DOCK4 or a non-target control (NTC), and the Rac1-GTP determined using a PBD assay after 48 h. *Top panel*, representative experiment. *Bottom panel*, densitometric analysis of Cdc42-GTP/total Cdc42. Results, relative to parental cells, are expressed as the mean ± S.E. (n = 3).

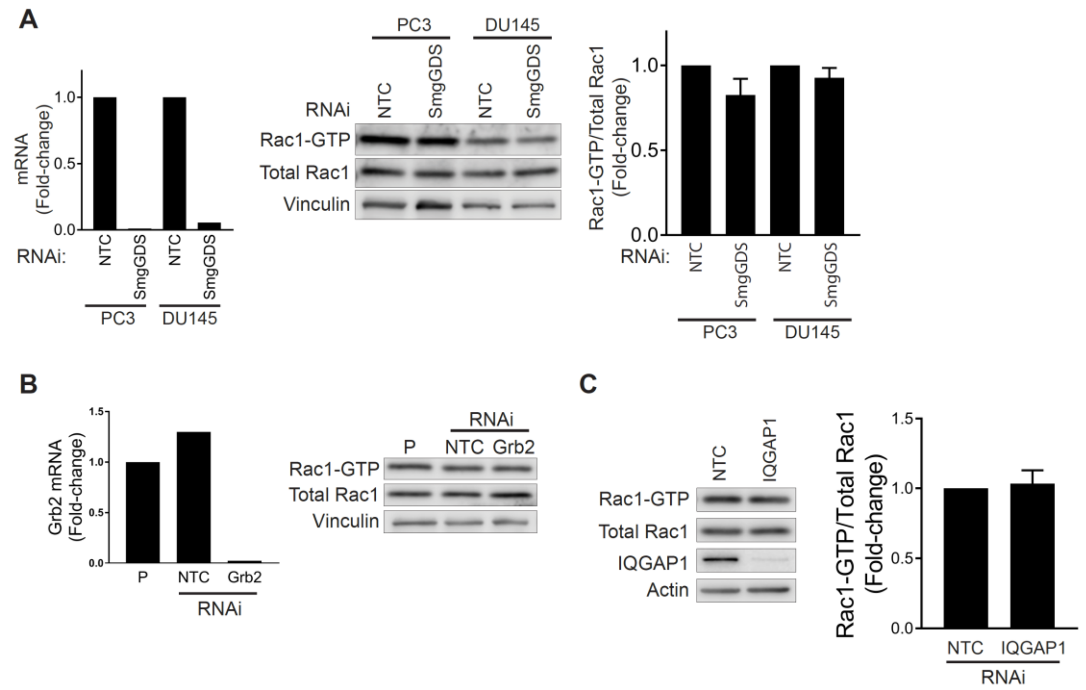

Figure S5. SmgGDS, Grb2 and IQGAP1 silencing have no effect on Rac1-GTP levels. (**A**) PC3 and DU145 cells were transfected with SmgGDS targeting or non-target control (NTC) RNAi duplexes for 48 h, including a 24 h serum starvation prior to the determination of Rac1-GTP levels using a PBD pull-down assay. *Left panel*, representative mRNA fold-change after RNAi. *Middle panel*, representative Rac1-GTP pulldown experiment. *Right panel*, densitometric analysis of Rac1-GTP levels normalized to total Rac1. Results, relative to NTC, are expressed as the mean ± S.E. (n = 3). (**B**) PC3 cells were transfected with Grb2 targeting or non-target control (NTC) RNAi duplexes for 48 hours, including a 24 h serum starvation prior to the determination of Rac1-GTP levels using a PBD pull-down assay. *Left panel*, representative mRNA fold-change after RNAi. *Right panel*, representative Rac1-GTP pulldown experiment. (**C**) PC3 cells were transfected with IQGAP1 or NTC RNAi duplexes for 48 h, including a 24 h serum starvation prior to the determination of Rac1-GTP levels. *Left panel*, representative Rac1-GTP pulldown experiment. *Right panel*, densitometric analysis of Rac1-GTP levels normalized to total Rac1. Results, relative to NTC, are expressed as the mean ± S.E. (n = 4).

Table S1. Proteomics of Rac1G15A binding proteins. See Excel file.

© 2020 by the authors. Licensee MDPI, Basel, Switzerland. This article is an open access article distributed under the terms and conditions of the Creative Commons Attribution (CC BY) license (http://creativecommons.org/licenses/by/4.0/).
