# Supplementary figures and images for: P-REX1-Independent, Calcium-Dependent RAC1 Hyperactivation in Prostate Cancer
Source: Cancers (Basel). 2020 Feb 19;12(2):480. doi: 10.3390/cancers12020480 (PMC7072377; doi:10.3390/cancers12020480)

Full blots from Figure S1A-C

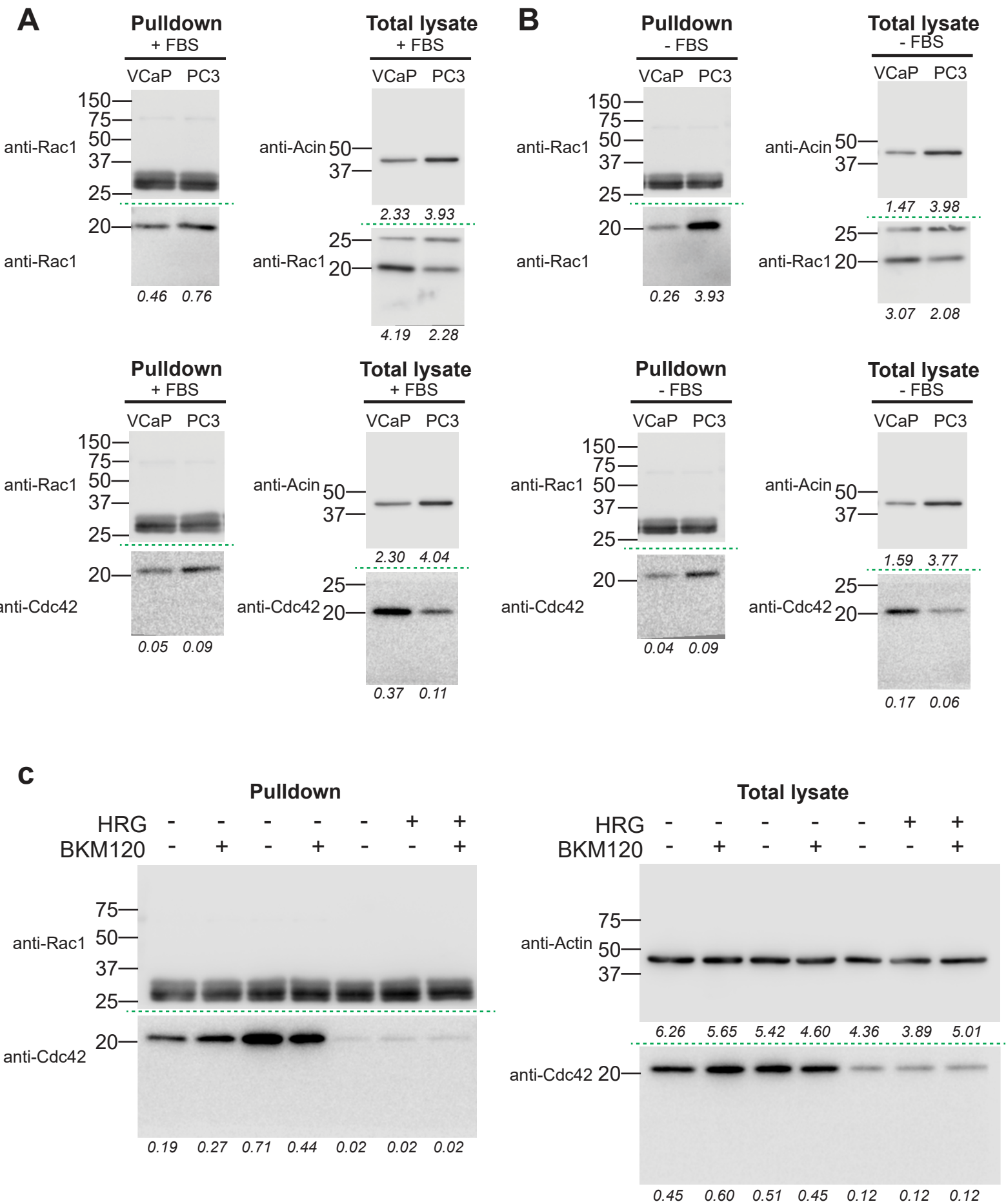

\* Green line, membrane was cut to blot with different antibodies.

Supplement: Supplementary file 1 [file cancers-12-00480-s001.zip › cancers-688668-SuppMaterials-final check/Figure S-1 A-C Westerns.pdf]

Full blots from Figure S1D-E

D

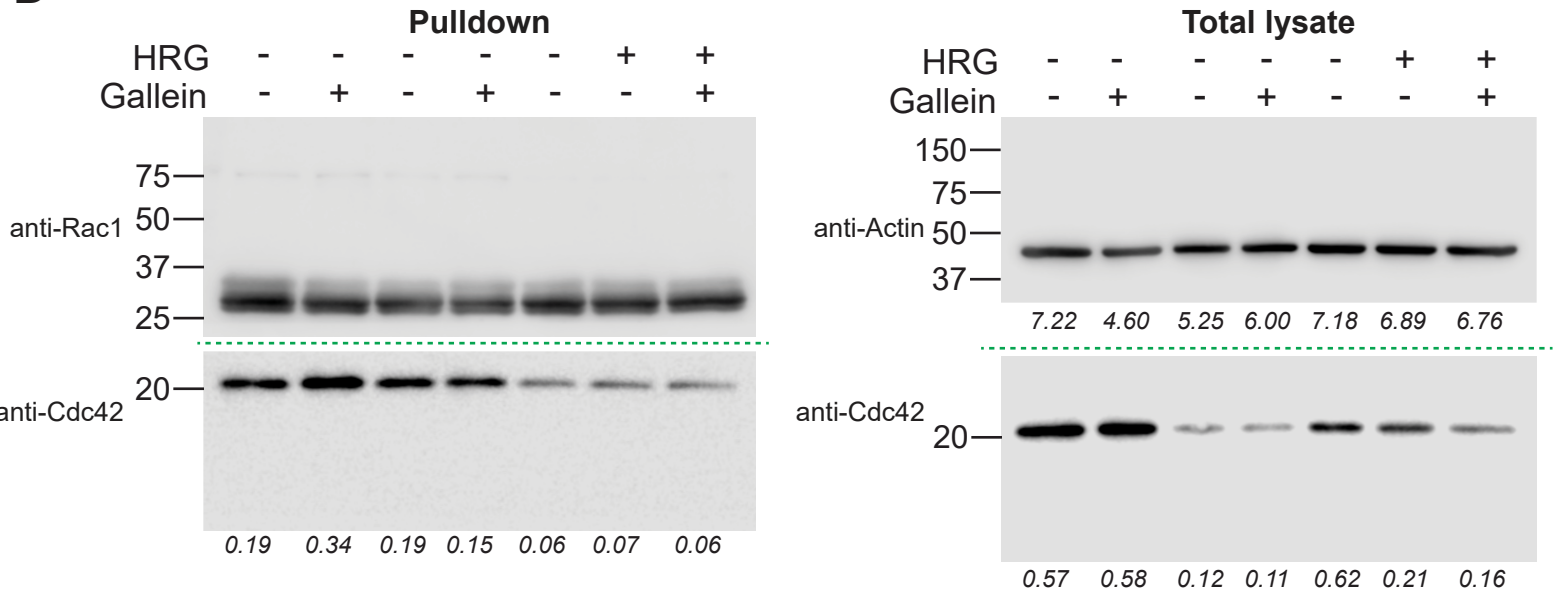

E

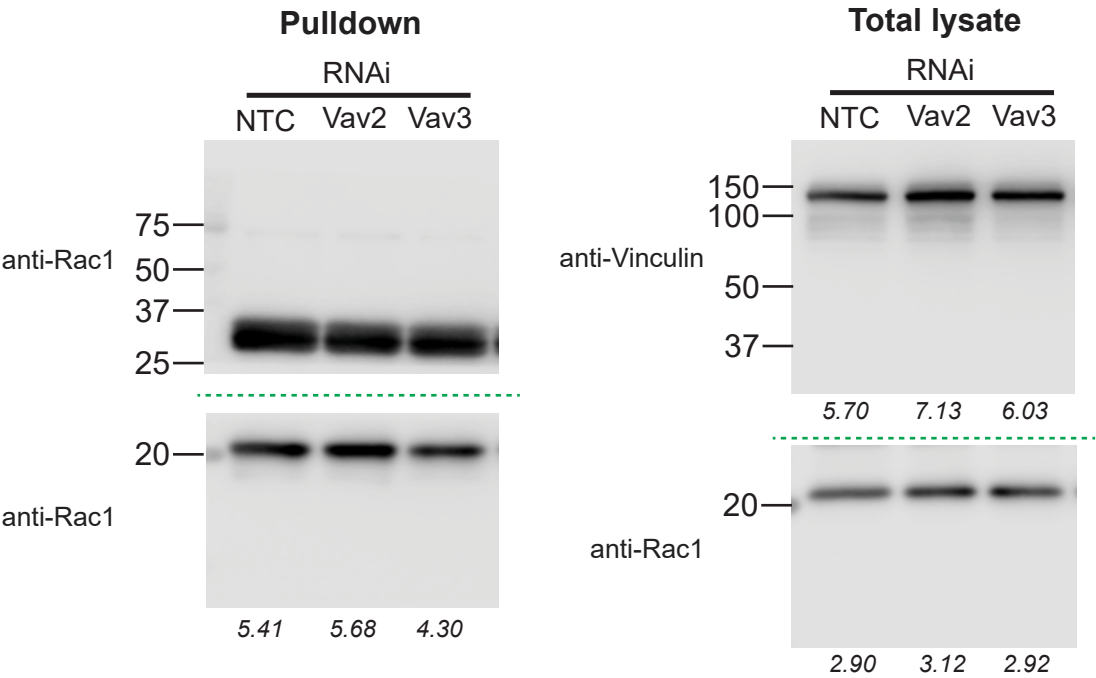

\* Green line, membrane was cut to blot with different antibodies.

Supplement: Supplementary file 1 [file cancers-12-00480-s001.zip › cancers-688668-SuppMaterials-final check/Figure S-1 D-E Westerns.pdf]

Full blots from Figure S2

A

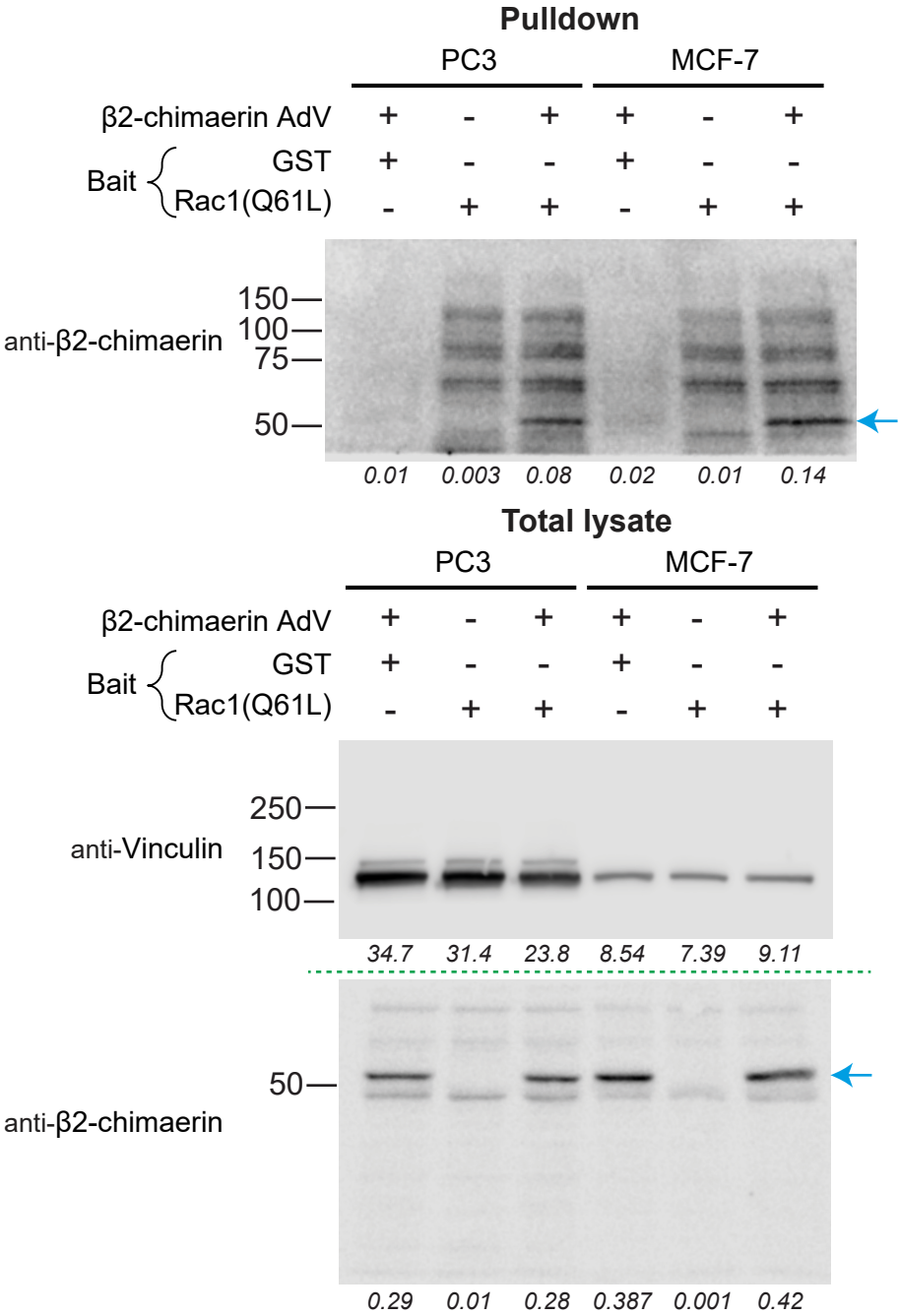

B

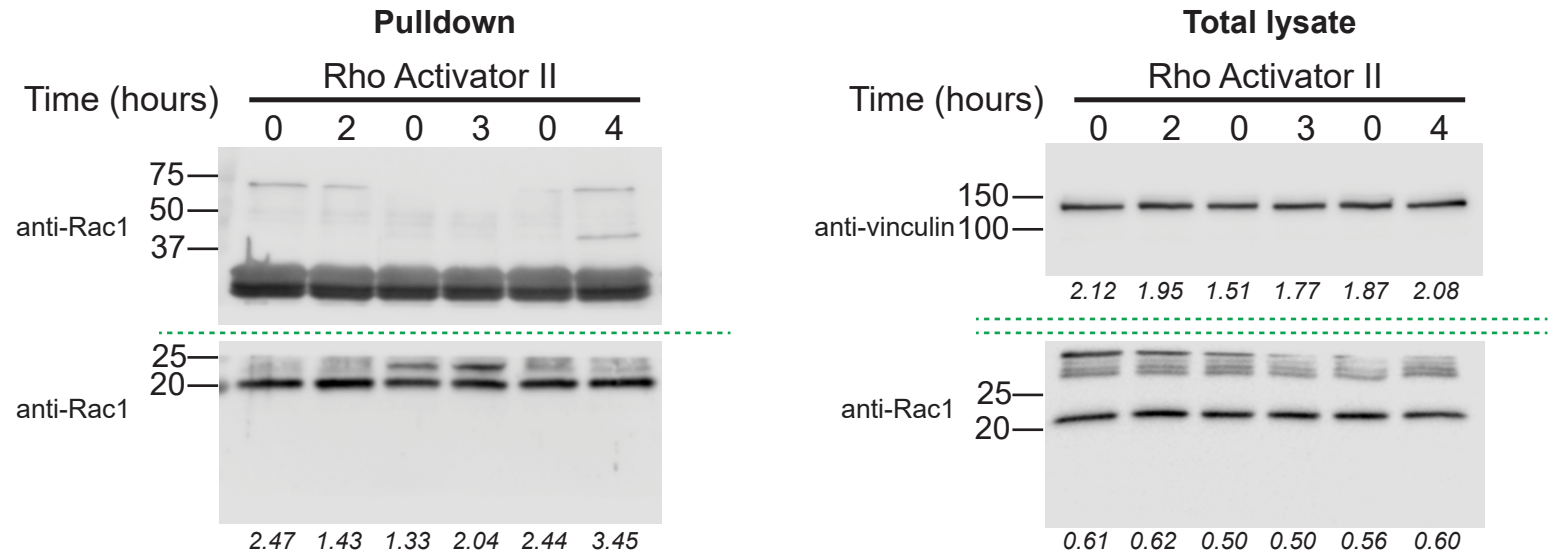

\* Green line, membrane was cut to blot with different antibodies.

Supplement: Supplementary file 1 [file cancers-12-00480-s001.zip › cancers-688668-SuppMaterials-final check/Figure S-2 Westerns.pdf]

# Full blots from Figure S4B

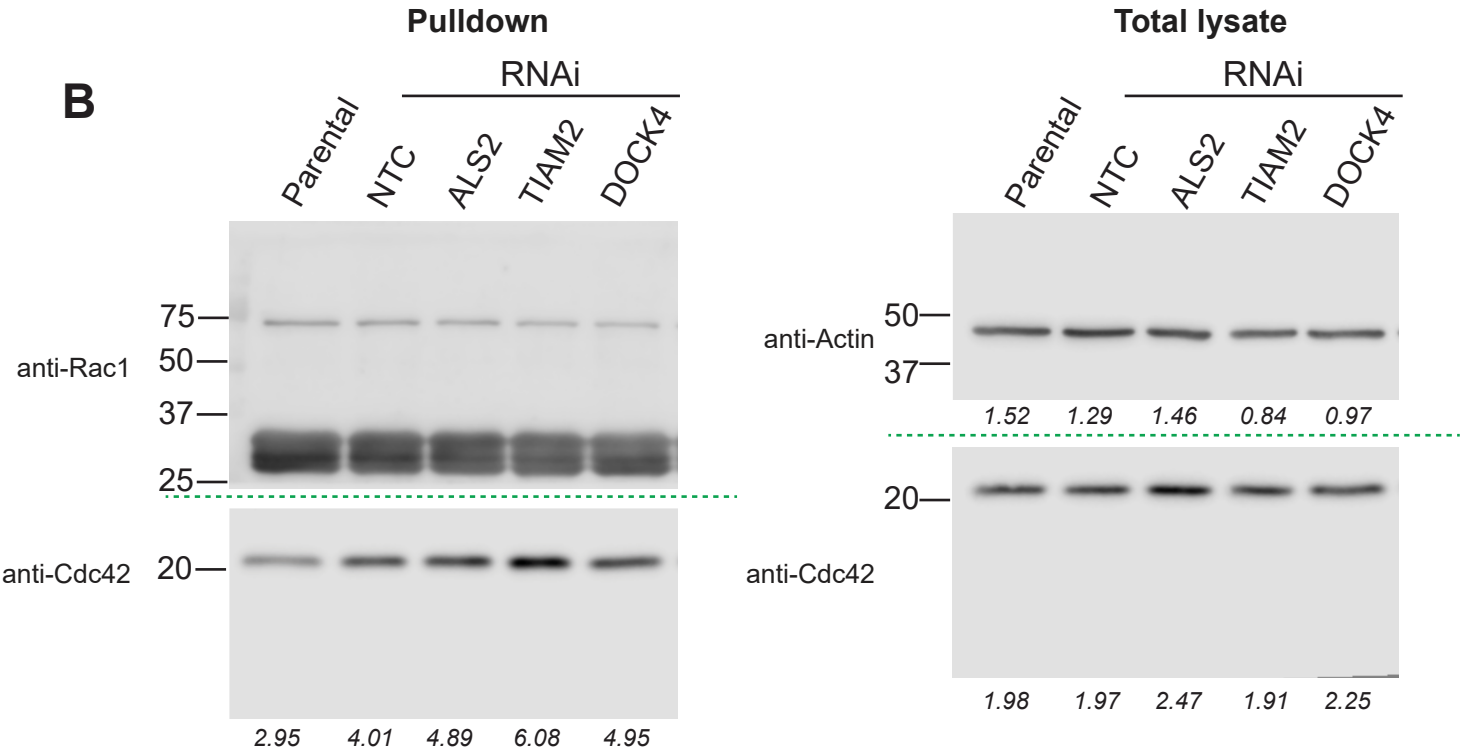

\* Green line, membrane was cut to blot with different antibodies.

Supplement: Supplementary file 1 [file cancers-12-00480-s001.zip › cancers-688668-SuppMaterials-final check/Figure S-4 Westerns.pdf]

# Full blots from Figure S5

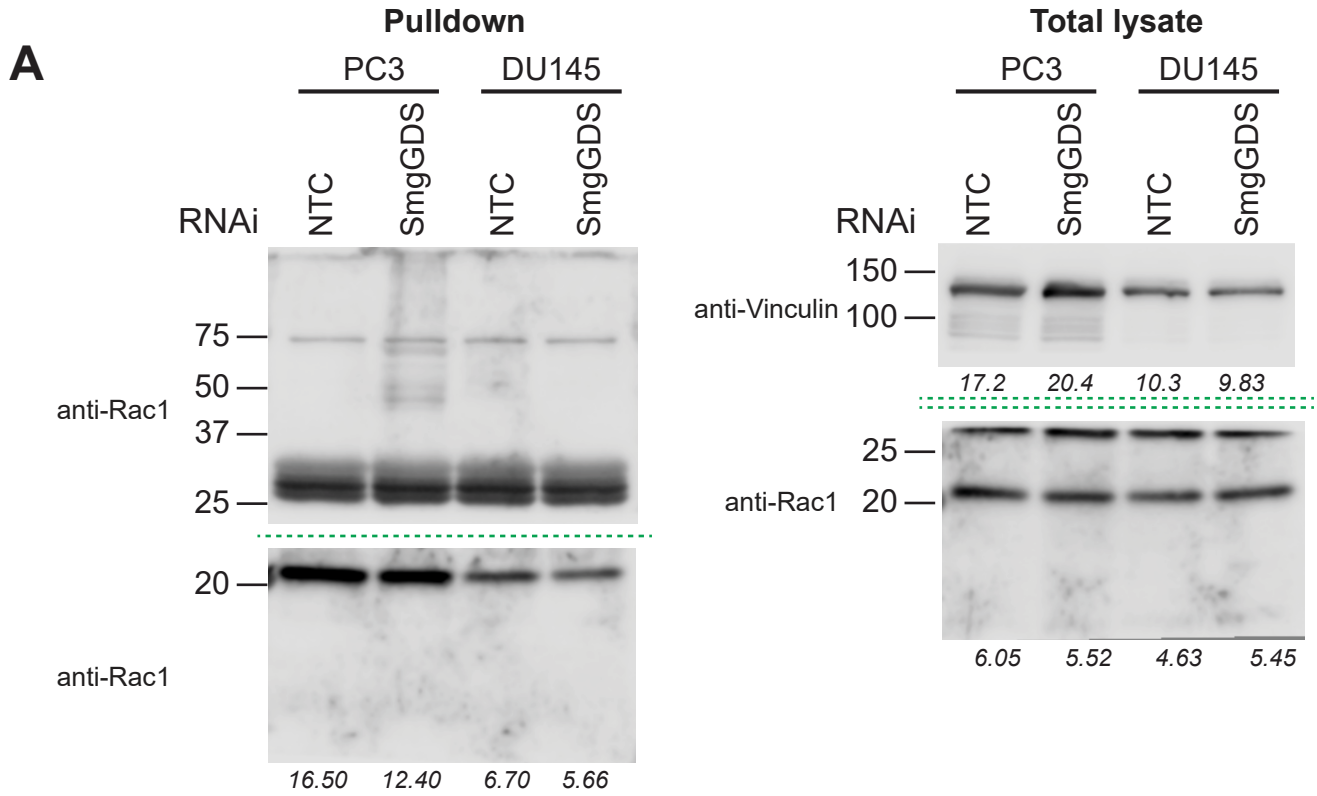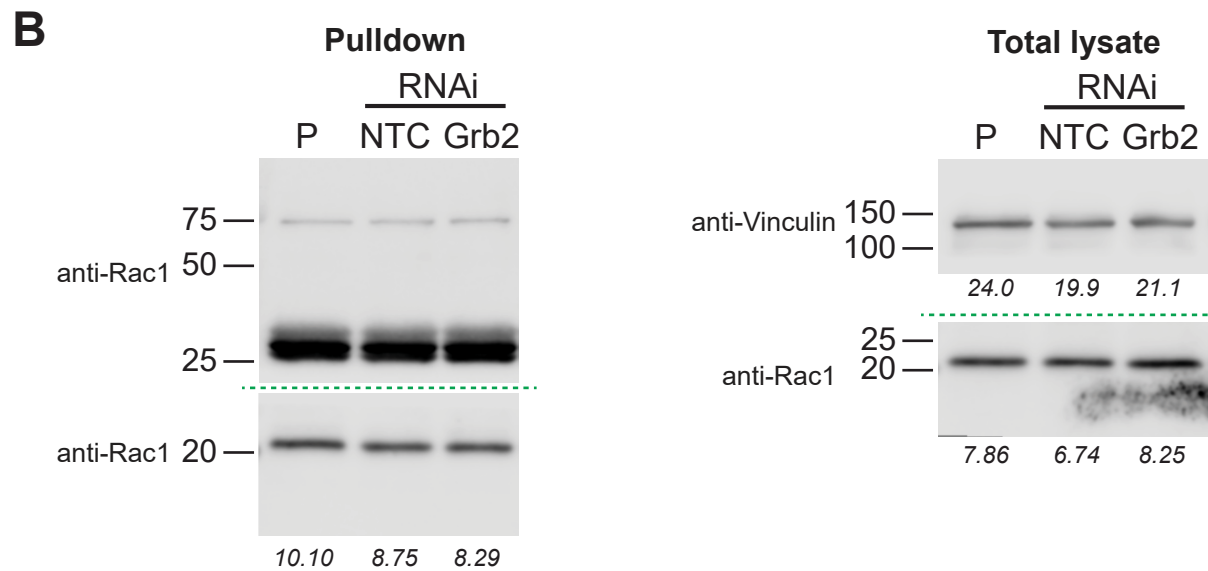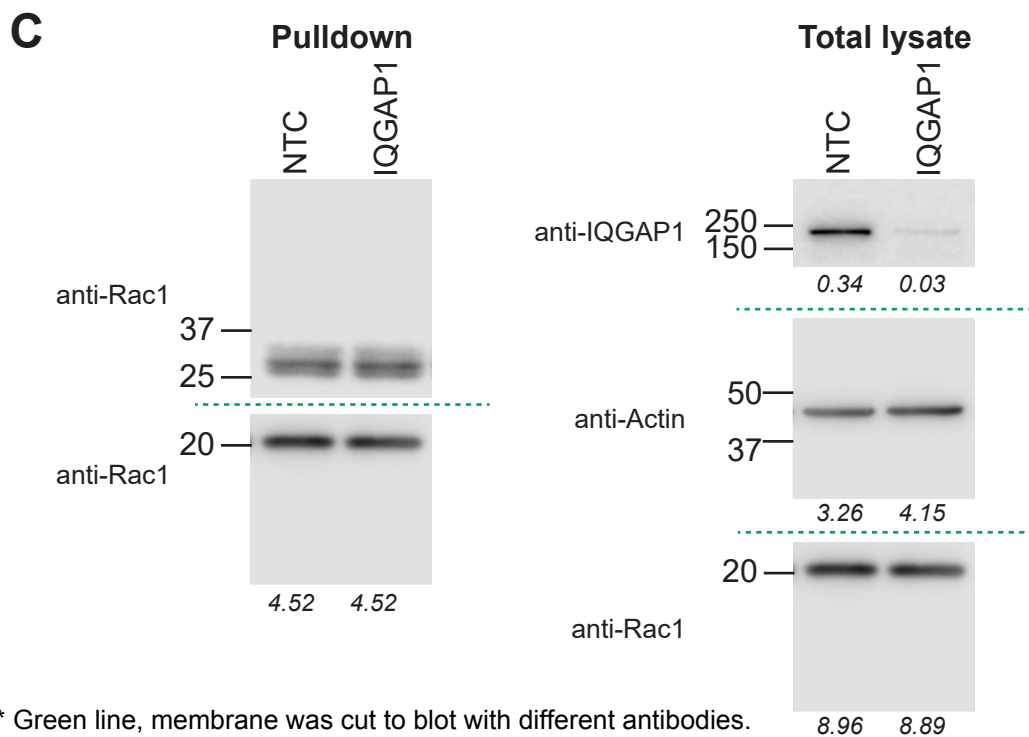

\* Green line, membrane was cut to blot with different antibodies.

Supplement: Supplementary file 1 [file cancers-12-00480-s001.zip › cancers-688668-SuppMaterials-final check/Figure S-5 Westerns.pdf]
